# Supplementary material for: Epidemiology, Risk Factors for Gastric Cancer and Surveillance of Premalignant Gastric Lesions: A Prospective Cohort Study of Central Saudi Arabia
Source: Curr Oncol. 2023 Sep 11;30(9):8338–51. doi: 10.3390/curroncol30090605 (PMC10528333; doi:10.3390/curroncol30090605)
Supplement: Supplementary file 1 [file curroncol-30-00605-s001.zip › curroncol-2490610-supplementary.pdf]

Epidemiology, Risk Factors for Gastric Cancer and Surveillance of Premalignant Gastric Lesions: a Prospective Cohort Study of Central Saudi Arabia

Supplementary materials: questionnaire to collect sociodemographic data and possible factors associated with gastric cancer (GC).

Survey Questionnaire: Factors that are possibly associated with GC included in the questionnaire were age, obesity (body mass index (BMI) > 30), smoking, a diet of salty preserved foods, income, education, and a family history of GC. The questions and answers were collected during history taking by the visit of the eligible participant in the study at the family medicine outpatient clinic.

SOCIODEMOGRAPHIC AND RISK FACTORS FOR GASTRIC CANCER QUESTIONNAIRE

Only for those who were positive in a high-sensitivity guaiac fecal occult blood test (HSgFOBT+) and had negative results in colonoscopy and had offered to undergo upper GI endoscopy.

Pt. No. ☐☐☐☐☐☐☐☐ *Seq. No. ☐☐

**Step No. Date

☐☐☐☐☐☐☐☐☐

The questions and answers

1. Age:

2. Gender: Female……..Male……Other…….

3. What is your nationality? Saudi…Non-Saudi… Ethnicity? Arab…… Afro-Arab………other….

4. Weight (kg)

5. Height (cm)

6. BMI:

A. Classify accordingly the following options:

a. Normal (18.5–25 kg/m^2)……….

b. Overweight (25–30 kg/m^2)…….

c. Obese (>30 kg/m^2)………………

7. Marital status:

- Single………..
- Married……..
- Divorced…....
- Widowed…….

8. Do you work for pay outside the home? Yes……No….

9. Occupation:

A. Check the box that best corresponds to your current work situation.

1. Working full time ....
2. Working part time ....
3. Not working and not looking for work ....
4. Unemployed and looking for work ....
5. Disabled or retired and not looking for work ....
6. Currently in school ....

B. Which of the following best characterizes your work type?

1. Freelancer

2. Salaried

4. Business

4. Owner

5. Unemployed

Other

10. Education level:

A. What is the highest level of education you have completed?

1. 12th grade or less ....
2. High school graduate or GED ....
3. Some college/AA degree/Technical school training ....
4. College graduate (BA or BS) ....
5. Graduate school degree: Master's or Doctorate degree (MD, PhD, JD)

B. Please classify according to the options below:

1. University, College……..……………………………….
2. Secondary education completed…..……………………
3. Primary education completed………………………….
4. Primary education not completed………………………

11. Housing

A. How many people are currently living in your household, including yourself? …………….

B. Please describe the home where you live.

(Check "Yes" or "No" for each question. Check "Yes" to all that apply.)

1. It is owned or being bought by you (or someone in the household). ......
2. It is rented for money by you (or someone in the household). ....
3. It is occupied without payment or money or rent. ....
4. I live with friends. ....
5. I live with family. ....
6. I have no permanent residence. ....
7. I live in a correctional facility (jail, prison). ....

12. Insurance

A. How do you pay for your health care?

1. Government funding (Medicaid, Medicare, ADAP, VA, Ryan White, etc.)
2. Private insurance ....
3. Self-pay, out of pocket

13. Income

A. What is your total combined family income for the past 12 months, before taxes, from all sources, wages, public assistance/benefits, help from relatives, alimony, and so on?

If you don't know your exact income, please estimate.

(Check one box)

1. Less than 10,000 sar ....
2. 10,000–20,000 sar ....

3. More than 20,000 sar ....

4. Don’t know ....

5. Chose not to answer……………………………………………………..

14. Smoking status

A. Do you currently smoke?

- Yes
- No

B. (only for current smokers) Based on your opinion/knowledge, please report which of the following products you are using

- Tobacco cigarettes
- Snus
- Electronic cigarettes
- Nicotine replacement therapies
- Oral smoking cessation medications (varenicline, bupropion)

C. Have you ever smoked in the past?

- Yes
- No

D. Please classify the participant accordingly to the following options:

a. Smoking status

b. Never smoked

c. Former smoker

d. Current smoker

15. Family history of GC

a. Yes

b. No

16. How many times have you practiced physical activity (at least 30 minutes)?

- One time per week……………………………
- 1–3 times per week……………………………
- More than 3 times per week……………………
- None……………………………………………….

17. Diet

A. What are the foods you eat daily? (Multiple options are available)

- Vinegar, lemon
- Apple, orange, grapefruit, date, grape, banana, other fruits not mentioned
- Tomato, cucumber, onion, carrot, garlic, cabbage, eggplant, other vegetables not mentioned
- Beans, lentils, chickpeas, lupine
- Coffee, tea
- Other

B. How many times per week you eat fresh fruits, vegetables, unprocessed wheat products?

- 1–3 times per week
- 4–7 times per weeks
- More than 7 times per week
- None

C. Do you eat fast food on a regular basis?

Yes…

No…

D. (only for those answered "yes"). How many times do you eat fast food on a weekly basis on average?..

E. Based on your opinion/knowledge, please score the health risk of each of the following products from 1 (lowest risk) to 10 (highest risk).

a. Fresh fruits, vegetables, unprocessed wheat products………………………………

b. Animal products, hot spices, canned and fermented foods…………………………

c. Nutritional salty preserved products…………………………………………………..

F. (only for users of nutritional salty preserved products) How many times per week?

- 1–3 times per week
- 4–7 times per weeks
- More than 7 times per week
- None

G. (only for users of salty preserved products) Based on your opinion/knowledge, please report which of the following products you are using

1. Salted fish (such as dried and salted cod or salted herring)

2. Salt-cured meat (such as beef bacon)

3. Pickles, cheeses, fermented beef sausages

4. Vegetables such as runner beans and cabbage

5. Other salted products……………….

E. (only for users of animal products, hot spices, canned and fermented foods) How many times per week?

- 1–3 times per week
- 4–7 times per weeks
- More than 7 times per week
- None

Thank you very much for completing this questionnaire.
